# Measles vaccination coverage estimates from surveys, clinic records, and immune markers in oral fluid and blood: a population-based cross-sectional study

**DOI:** 10.1186/1471-2458-13-1211

**Published:** 2013-12-20

**Authors:** Kyla T Hayford, Mohammed S Shomik, Hassan M Al-Emran, William J Moss, David Bishai, Orin S Levine

**Affiliations:** 1 Department of International Health, International Vaccine Access Center (IVAC), Johns Hopkins Bloomberg School of Public Health, 855 N. Wolfe Street, Suite 600, Baltimore, MD 21205, USA; 2 International Centre for Diarrhoeal Disease Research, Bangladesh (icddr,b), Mohakhali, Dhaka 1212, Bangladesh; 3 Child Health Research Foundation (CHRF), Dhaka Shishu Hospital, Dhaka 1212, Bangladesh; 4 Department of Epidemiology, Johns Hopkins Bloomberg School of Public Health, 615 N. Wolfe Street, Baltimore, MD 21205, USA; 5 Department of Population, Family and Reproductive Health, Johns Hopkins Bloomberg School of Public Health, Baltimore, 615 N. Wolfe Street, Baltimore, MD 21205, USA

**Keywords:** Immunization, Vaccination, Immune marker, Surveillance, Measles, Oral fluid, Vaccination card, Maternal report, Bangladesh

## Abstract

**Background:**

Recent outbreaks of measles and polio in low-income countries illustrate that conventional methods for estimating vaccination coverage do not adequately identify susceptible children. Immune markers of protection against vaccine-preventable diseases in oral fluid (OF) or blood may generate more accurate measures of effective vaccination history, but questions remain about whether antibody surveys are feasible and informative tools for monitoring immunization program performance compared to conventional vaccination coverage indicators. This study compares six indicators of measles vaccination status, including immune markers in oral fluid and blood, from children in rural Bangladesh and evaluates the implications of using each indicator to estimate measles vaccination coverage.

**Methods:**

A cross-sectional population-based study of children ages 12–16 months in Mirzapur, Bangladesh, ascertained measles vaccination (MCV1) history from conventional indicators: maternal report, vaccination card records, ‘card + history’ and EPI clinic records. Oral fluid from all participants (n = 1226) and blood from a subset (n = 342) were tested for measles IgG antibodies as indicators of MCV1 history and compared to conventional MCV1 coverage indicators.

**Results:**

Maternal report yielded the highest MCV1 coverage estimates (90.8%), followed by EPI records (88.6%), and card + history (84.2%). Seroprotection against measles by OF (57.3%) was significantly lower than other indicators, even after adjusting for incomplete seroconversion and assay performance (71.5%). Among children with blood results, 88.6% were seroprotected, which was significantly higher than coverage by card + history and OF serostatus but consistent with coverage by maternal report and EPI records. Children with vaccination cards or EPI records were more likely to have a history of receiving MCV1 than those without cards or records. Despite similar MCV1 coverage estimates across most indicators, within-child agreement was poor for all indicators.

**Conclusions:**

Measles IgG antibodies in OF was not a suitable immune marker for monitoring measles vaccination coverage in this setting. Because agreement between conventional MCV1 indicators was mediocre, immune marker surveillance with blood samples could be used to validate conventional MCV1 indicators and generate adjusted results that can be compared across indicators.

## Background

Surveillance of vaccination coverage is important for disease control, monitoring health system performance, and as a benchmark for progress toward Millennium Development Goal 4 on reducing child mortality [1-3]. However, conventional vaccination coverage estimates based on administrative records or household surveys are at risk of unintentional and intentional errors of omission, recording and recall [4-11]. Financial incentives for improving coverage can exacerbate these biases [11]. Administrative records from Expanded Programme on Immunisation (EPI) vaccination clinics generate vaccination coverage estimates in real-time with little added cost, but can suffer significant biases if reporting is incomplete or the eligible population size is not accurately measured [10,12-14]. Household surveys, such as the Demographic and Health Surveys (DHS) or Multiple Indicator Cluster Survey (MICS), generate three vaccination coverage indicators based on: 1) maternal report, 2) household-retained vaccination cards, or 3) a composite ‘card + history’ indicator, which uses vaccination card data or, if not available, maternal report. The ‘card + history’ indicator is regarded as the best available data source balancing accuracy with completeness but surveys are expensive and do not generate coverage estimates in real-time [6,7,15-17]. Consequently, comparisons of vaccination coverage using different indicators are often invalid [4-6,10-12].

Recent outbreaks of measles in low-income countries highlight that conventional vaccination coverage indicators do not adequately identify susceptible children. There is increasing interest in immune marker surveillance to monitor immunization programs and estimate population immunity attributable to vaccination [18-20]. However, immune marker surveillance with blood or oral fluid (OF) samples pose other errors and biases related to sample collection, storage, testing and interpretation of results as well as financial and logistical challenges in the field [21]. In this study, we compared six indicators of measles vaccination status, including immune markers of measles immunity in oral fluid and blood, from young children in rural Bangladesh and evaluated the implications of using each indicator to estimate measles vaccination (MCV1) coverage.

## Methods

The study was conducted among children aged 12–16 months residing in the Mirzapur Demographic Surveillance System (DSS), a rural area 60 km north of Dhaka, Bangladesh. The DSS is comprised of ~240,000 individuals living in 58,300 households visited every 4 months to update births, deaths and migrations. A stratified random sample of 1450 children was selected from the eight *unions* (administrative unit similar to a county) of the DSS with probability of proportional to eligible population size in each union. Ethics committees at the International Centre for Diarrhoeal Disease Research, Bangladesh (icddr,b) and the Johns Hopkins Bloomberg School of Public Health approved the study and written permission from the participants’ guardian was obtained prior to enrollment.

Data collection took place in three stages: a caregiver survey and oral fluid collection in the household, review of EPI clinic records, and blood collection from a subset of children in four of the eight *unions*. A wealth index was generated using principal component analysis based on household assets [22]. Record books from EPI clinics were obtained and records were matched to enrolled children based on EPI registration IDs from their vaccination cards or by matching at least three variables: child name, birth date (±3 days), father’s name or grandfather’s name.

OF samples were obtained by rubbing a foam swab (Oracol; Malvern Medical Developments, Worcester, UK) along the child’s gumline for 1–1.5 minutes as described elsewhere [23]. OF samples were transported in cold boxes within 4 hours to the local laboratory where 1 mL of transport buffer was added, centrifuged at 2000 rpm for five minutes, and the supernatant was stored at -20°C until testing. OF samples were tested for antibodies to measles virus using a measles virus-specific IgG capture enzyme immunoassay (ELISA) (Microimmune Ltd, Middlesesex, UK), which was validated for use with OF samples with a reported sensitivity of 93% and specificity of 98% compared to serum [24]. OF samples were categorized as positive, equivocal or negative; equivocal samples were retested and, if equivocal again, were considered positive in binary analyses. To identify poor quality OF samples, specimens testing negative for measles IgG were tested for total IgG antibodies by ELISA (Bethyl Laboratories, Montgomery, TX) and excluded from the analysis if less than 125 ng/mL.

Peripheral blood samples were collected by trained phlebotomists from all enrolled children with parental permission within one month of OF collection in four of eight *unions.* Serum samples were extracted and stored at -20°C until tested for measles IgG antibodies (Enzygnost ELISA, Siemens, Germany) with a reported sensitivity and specificity of 99.6% and 100%, respectively [25]. Serum samples were categorized as negative, equivocal or positive as recommended by the manufacturer. Serum samples testing equivocal were re-tested and, if equivocal again, were categorized as positive.

Up to six indicators of MCV1 history were ascertained for each child: 1) maternal report of MCV1 based on recall questions modified from the DHS [26]; 2) card record of MCV1 based on evidence and dates of MCV1 receipt on the child’s household retained vaccination card, if available; 3) ‘card + history’; 4) EPI record of MCV1 based on dates and evidence of MCV1 abstracted from EPI clinic record books; 5) protective levels of measles IgG antibodies in OF; and 6) protective levels of measles IgG antibodies in blood. MCV1 coverage by OF was adjusted for assay sensitivity and specificity using following equation: P_adjusted_ = (P_observed_ – (1-specificity))/(1 – [(1-specificity) + (1-sensitivity)]). Seroprotection in OF and blood was assumed to be vaccine induced because maternal antibodies should have been non-detectable is this age group and the last measles outbreak in the district occurred more than a year before study participants were born according to the WHO measles surveillance laboratory in Dhaka, Bangladesh [27,28]. Study participants were not age eligible for the 2010 MCV campaign.

Categorical variables were compared using chi-square tests or Fisher’s exact test and continuous variables with non-normal distributions were compared using the Wilcoxon rank sum test. MCV1 coverage estimates were generated with exact binomial confidence intervals and compared using McNemar’s test for paired samples. Bivariate logistic and log binomial models were used to calculate odds ratios and prevalence ratios, respectively. Sensitivity, specificity and kappa were calculated to evaluate within-child agreement of indicators. Analysis was conducted in Stata 11 (StataCorp LP, College Park, TX, USA).

## Results

**Figure 1:**
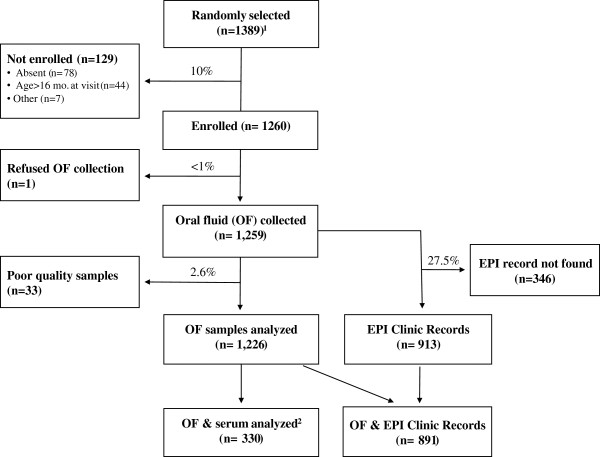
**Enrollment chart. **^1^ 61 children selected from the database were not included because they migrated out of DSS before initiation of study. ^2^ Blood samples were collected and analyzed from 342 children. Paired OF and blood samples were available for 330 children.

1450 children were randomly selected from the DSS database, of whom 1389 were living in the DSS area at the time of the study. Of these children, 1260 (89.8%) were enrolled in the study from September 2010 to January 2011 (Figure 1, Additional file 1: Table S1). The parent of one child refused OF collection and 33 OF samples were excluded for poor quality (total IgG antibodies <125 ng/mL), resulting in 1226 children in the primary analysis. Vaccination records from EPI record books were found for 913 (72.5%) children, of whom 891 had an adequate OF sample. Blood was collected from 342 children with a 7.7% refusal rate. Of children with blood sample results, 311 (91%) had adequate OF samples and 268 (78%) had EPI clinic records.

No differences in the age, sex, asset quintile, household size, or education level of the father was observed for enrolled versus non-enrolled children, but mothers with more education (unadjusted OR 0.94 for each additional year; 95% CI: 0.89, 0.99), working mothers (OR 0.38; CI: 0.21, 0.68), and families that had moved at least once since 2005 (OR 0.47; CI: 0.32, 0.68) were significantly less likely to be enrolled.

### Factors associated with MCV1 history

The mean age of enrolled children was 14.5 months with an equal proportion of males and females (Table 1). Using the card + history indicator as the best available data source, MCV1 coverage did not differ by age or sex, but children taken by family members other than the mother for vaccination were less likely to have received MCV1. Higher socioeconomic status (SES) based on asset quintiles was associated with receipt of MCV1. Children vaccinated against measles were more likely to be born in a hospital or clinic, have a skilled birth attendant or doctor assist in their delivery, and have more antenatal and postnatal care visits than unvaccinated children (Table 1).

**Table 1:** Characteristics of enrolled children and factors associated with receipt of MCV1

			**Card + History indicator**		
		**Overall**	**Vaccinated**	**Unvaccinated**	
	**Frequency**	**%**	**(1033 children)**	**(193 children)**	**p-value**^**1**^
Age at enrollment, mean (SD)	14.5 (1.4)		14.5 (1.5)	14.5 (1.4)	0.930
Male	628	51.2	534 (51.7)	94 (48.7)	0.446
Father’s education, median [IQR] (years)	7 [5, 9]		7 [5, 10]	5 [0, 9]	**<0.001**
Mother’s education, median [IQR](years)	7 [4, 9]		7 [5, 9]	5 [2, 8]	**<0.001**
Mother had job in last 3 years	154	12.6	116 (11.2)	38 (19.7)	**0.001**
Asset quintile (2010)^2^					
1 - poorest	246	20.1	192 (18.6)	54 (28.0)	**0.001**
2	250	20.4	202 (19.6)	48 (24.9)	
3	246	20.1	208 (20.1)	38 (19.7)	
4	243	19.8	215 (20.8)	28 (14.5)	
5 - wealthiest	241	19.7	216 (20.9)	25 (13.0)	
Retained child's vaccination card	1019	83.1	872 (84.4)	147 (76.1)	**0.005**
Mother responsible for bringing child for vaccinations	1060	86.5	903 (87.4)	159 (79.9)	**0.006**
Location of child’s delivery^3^					
Hospital or clinic	460	37.5	402 (38.9)	58 (30.0)	**0.034**
House (own, family or friend’s home)	752	61.3	621 (60.1)	131 (67.9)	
Person assisting with child's delivery^3^					
Qualified doctor	283	23.1	253 (24.5)	30 (15.4)	**0.029**
Skilled birth attendant	209	17.0	178 (17.2)	31 (16.1)	
Traditional birth attendant	73	54.9	554 (53.6)	119 (61.7)	
Other (mother, informal health worker, alone)	47	3.8	38 (3.7)	9 (4.7)	
Antenatal care visits^3^					
None	320	26.1	251 (24.3)	69 (35.8)	**<0.001**
1	246	20.1	203 (19.6)	43 (22.3)	
2 or more	645	52.6	568 (55.0)	77 (39.9)	
Postnatal care visits^3^					
None	645	52.6	525 (50.8)	120 (62.2)	**0.007**
1	412	33.6	364 (35.2)	48 (24.9)	
2 or more	155	12.6	134 (13.0)	21 (10.9)	

^1^Chi-square or Fisher’s exact test for categorical variables. Wilcoxon rank sum test for non-normal continuous variables.

^2^Asset quintiles calculated using principal component analysis from household asset data.

^3^Data on child’s delivery (n = 14), antenatal care (n = 15) and postnatal care (n = 14) not available for small number of children.

### MCV1 Coverage by indicator

**Figure 2:**
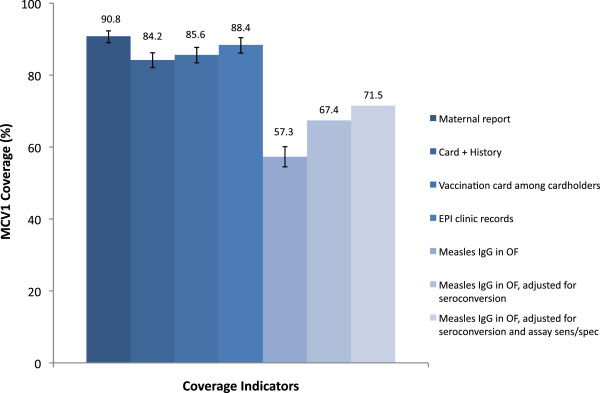
**Overall MCV1 Coverage by Indicator.** Note: MCV1 coverage based on all available data and sample size varies by indicator. ‘Oral fluid adjusted for seroconversion’ assumes 85% of vaccinated children seroconverted. ‘Oral fluid, adjusted for seroconversion and assay sensitivity/specificity’ assumes 85% seroconversion and adjusts for the sensitivity (93%) and specificity (98%) of the assays, as reported by the manufacturer.

**Figure 3:**
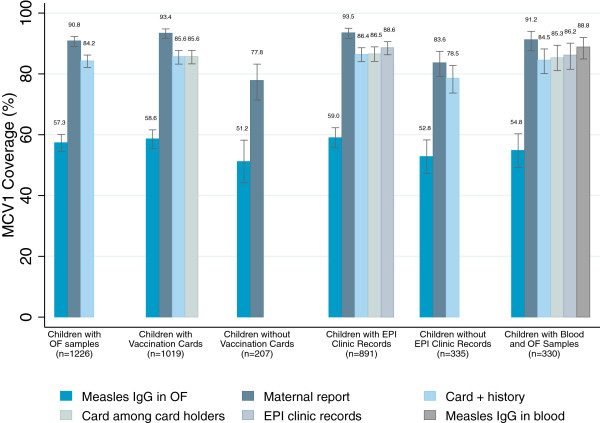
**Measles vaccination coverage by indicator among subgroups of children with complete data.** Note: Coverage from EPI records is not representative of the population because ~27% of records could not be located. Children with blood samples represent a subset of study population (n = 330 except for EPI clinic records where n = 268).

Maternal report yielded the highest coverage estimates (90.8% CI: 89.0, 92.3), followed by EPI records (88.6%; CI: 86.3, 90.6), card records among card holders (85.6%; CI: 83.3, 87.7), and card + history (84.2%; CI: 82.1, 86.2). Coverage of protective levels of measles IgG antibodies in OF (57.3%; CI: 54.5, 60.1) was significantly lower than conventional indicators (Figure 2). Two adjustments were made to the raw OF prevalence to account for differences between vaccination history and immunologic status. Assuming 85% seroconversion among children ages 9–10 months, MCV1 coverage by OF was 67.4% (i.e. 57.3/0.85 = 67.4%) [29]. A subsequent adjustment accounting for the reported sensitivity and specificity of the assay generated a final adjusted MCV1 coverage estimate of 71.5% by OF, which remained statistically significantly lower than coverage based on all conventional MCV1 indicators (McNemar’s test, p < 0.001 for all pairwise tests) (Figure 3). For the 342 children with blood results, 83.0% had protective levels of measles IgG antibodies, 11.2% were susceptible and 5.8% had equivocal results, which were categorized as positive in binary analyses for an overall prevalence of 88.6% (CI: 85.2, 92.0). For children with both blood and OF samples, MCV1 coverage by blood serostatus was significantly higher than by card + history (84.5%, McNemar’s test p = 0.038) but non-significant differences were detected for maternal report (90.9%, p = 0.10) and EPI records (86.0%, p = 0.16).

To account for missing data for several indicators, MCV1 coverage indicators were compared within subgroups of children who had complete data (Figure 3). Within each subgroup, MCV1 coverage based on OF was significantly lower than all other coverage indicators (p < 0.001 for all pairwise tests). MCV1 coverage by maternal report was consistently higher than other indicators (p < 0.001 for all pairwise tests) except among those with both blood and OF samples. To capture systematic differences between indicators, prevalence ratios comparing MCV1 coverage for each pair of indicators were generated to show the observed over- or under-estimation in MCV1 coverage estimates (Table 2).

**Table 2:** Prevalence ratios for MCV1 coverage to adjust for differences between indicators: an approach to comparing vaccination coverage indicators

***Comparison indicator***	***Prevalence ratio [95% CI]***			
***Reference indicator***	**Maternal report**	**Card + History**	**EPI records**	**Serum**
**Serum**	1.026	**0.954**	0.952	–
	[0.998, 1.056]	**[0.914, 0.996]**	[0.893, 1.015]	
	n = 342	n = 342	n = 278	
**EPI records**	**1.057**	0.976	–	
	**[1.024, 1.088]**	[0.940, 1.013]		
	n = 891	n = 891		
**Card + History**	**1.077**	–		
	**[1.059, 1.096]**			
	n = 1226			
**Maternal report**	–			

Note: Estimates are prevalence ratios [95% confidence intervals] to estimate the degree of over- or underestimation for each method used. The prevalence ratio based on comparison indicator (row) divided by reference indicator (column). E.g. the maternal report indicator generates an MCV1 coverage estimate 7.7% higher (PrR: 1.077 = 0.9078/0.8426) than coverage estimated by card + history. **Bold** indicates statistical significance with a 5% two-sided alpha. Results from this study are presented as an example of a tool governments or EPI programs could develop to compare vaccination coverage estimates from different indicators.

83% of parents showed their child’s vaccination card at the interview, of which 85.6% (CI: 83.4, 87.7) had evidence of MCV1 on the card (Figure 3). Children with vaccination cards were significantly more likely to have received MCV1 than children without cards by all conventional indicators and blood serostatus (maternal report: 93.4% (card) vs. 77.8% (no card), p < 0.001; EPI records: 86.9% vs. 100%, p = 0.006; OF: 58.6% vs. 51.2%, p = 0.054; blood: 90.5% vs. 77.8%, p = 0.02). EPI records were located for 72.5% of enrolled children. Children with available EPI records were more likely to be younger, have lower asset scores, not moved since the child was born, and retained the child’s vaccination card than children whose records were not located (Table 3). Children with EPI records had significantly higher MCV1 coverage by maternal report (93.5% with EPI records vs. 83.6% without EPI records, p < 0.001), card + history (86.4% vs. 78.5%, p = 0.001), and blood serostatus (90.7% vs. 80.6%, p = 0.041).

**Table 3:** Characteristics of children with and without EPI clinic records

	**EPI records found (n = 913)**		**EPI records not found (n = 346)**		
	**Frequency**	**%**	**Frequency**	**%**	**p-value**^**1**^
Age at enrollment (months), mean (SD)	13.8 (1.4)		14.0 (1.4)		**0.026**
Male	456	50.0	186	53.8	0.227
Father’s Education, in years, median (IQR)	6 [4, 9]		8 [5, 10]		**<0.001**
Moved since child’s birth	327	35.8	163	47.1	**<0.001**
Asset quintile					
1 (poorest)	198	21.7	55	15.9	**0.001**
2	191	20.9	61	17.6	
3	177	19.4	75	21.7	
4	189	20.7	62	17.9	
5 (wealthiest)	158	17.3	93	26.9	
Retained vaccination card	800	87.6	246	71.1	**<0.001**
History of MCV1 by maternal report	853	93.4	287	83.0	**<0.001**
History of MCV1 by card + history	790	87	270	78	**<0.001**
Fully vaccinated, by card + history	657	72.0	187	54.0	**<0.001**
Union					
Ajgana	139	15.2	67	19.4	**<0.001**
Bahuria	146	16.0	25	7.2	
Banail	104	11.4	14	4.0	
Bhatgram	108	11.8	20	5.8	
Gorai^2^	98	10.7	114	33.0	
Jamurki	101	11.1	45	13.0	
Uarsi	116	12.7	12	3.5	
Mirzapur	101	11.1	49	14.1	

^1^Chi square test for categorical variables. Wilcoxon rank sum test for non-normally distributed continuous variables.

^2^Record books from two EPI clinics in Gorai were not available.

Higher asset quintiles were associated with higher MCV1 coverage for all indicators except card records and EPI records with the most substantial gap between the bottom 20% and top 80% of asset scores (Table 4). The relative odds of MCV1 history by maternal report was 2.41 times greater than for children in the top 80% of asset scores (CI: 1.59, 3.65). Restricting analyses to children with blood data attenuated differences across asset quintiles for all indicators except coverage based on blood serostatus. Children in the highest 80% of asset scores had significantly higher odds of having protective levels of measles IgG antibodies in blood than children in the lowest 20% (OR 2.48; CI: 1.23, 5.01), whereas there was no difference for maternal report (OR 1.77; CI: 0.79, 3.94), card + history (OR 1.31; CI: 0.67, 2.57), or EPI records (OR 0.90; CI: 0.40, 1.99) (Table 4). Significant differences in measles serostatus remained between the poorest quintile and other quintiles after adjusting for MCV1 history (card + history) (OR 2.74; CI: 1.10, 6.90).

**Table 4:** Association between indicators of MCV1 coverage and socioeconomic status

**All children in study (n = 1226)**							
	**Asset quintile**					**Total**	**p-value**^**2**^
	**1 – poorest**	**2**	**3**	**4**	**5 - wealthiest**		
Maternal recall (n = 1226)^1^	83.7	88.0	93.1	95.1	94.2	90.8	
	(78.4, 88.1)	(83.3, 91.8)	(89.2, 95.9)	(91.5, 97.4)	(90.4, 96.8)	(89.0, 92.3)	**<0.001**
Card + History (n = 1226)^1^	78.0	80.8	84.6	88.5	89.6	84.2	**0.003**
	(72.3, 83.1)	(75.4, 85.5)	(79.4, 88.8)	(83.8, 92.2)	(85.1, 93.2)	(82.1, 86.2)	
Card record (n = 1046)	82.8	84.7	84.8	86.7	89.4	85.7	
	(77.0, 87.6)	(79.1, 89.3)	(79.4, 89.2)	(81.2, 91.1)	(84.4, 93.2)	(83.4, 87.7)	0.135
Oral fluid (n = 1226)^1^	49.2	55.2	60.6	61.7	60.2	57.3	
	(42.8, 55.6)	(48.8, 61.5)	(54.2, 66.7)	(55.3, 67.9)	(53.7, 66.4)	(54.5, 60.1)	**0.004**
EPI records (n = 913)	87.9	89.0	88.7	91.0	84.8	88.4	
	(82.5, 92.1)	(83.7, 93.1)	(83.1, 93.0)	(86.0, 94.6)	(78.2, 90.0)	(86.1, 90.4)	0.25
*% of EPI records found*	78%	76%	70%	75%	63%	73%	
**Children with blood samples (n = 342)**							
	**1 - poorest**	**2**	**3**	**4**	**5 - wealthiest**	**Total**	**p-value**^**2**^
Maternal recall (n = 342)	86.8	87.3	92.7	93.8	94.7	90.9	
	(77.1, 93.5)	(77.3, 94.0)	(82.4, 98.0)	(84.8, 98.3)	(87.1, 98.5)	(87.4, 93.8)	0.163
Card + History (n = 342)	81.6	74.6	85.5	87.5	93.4	84.5	
	(71.0, 89.5)	(62.9, 84.2)	(73.3, 93.5)	(76.8, 94.4)	(85.3, 97.8)	(80.2, 88.2)	0.425
Card record (n = 294)^1^	82.1	78.3	86.3	88.0	92.4	85.4	
	(70.8, 90.4)	(65.8, 87.9)	(73.7, 94.3)	(75.7, 95.5)	(83.2, 97.5)	(80.8, 89.2)	0.388
Oral fluid (n = 330)^1^	50.7	52.8	54.7	61.7	55.4	54.8	
	(38.7, 62.6)	(40.6, 64.9)	(40.4, 68.4)	(48.2, 73.9)	(43.4, 67.0)	(49.3, 60.3)	0.418
Serum (n = 342)	80.2	90.1	89.1	90.6	93.4	88.6	
	(69.5, 88.5)	(80.7, 95.9)	(77.8, 95.9)	(85.3, 98.9)	(85.3, 97.8)	(85.2, 92.0)	**0.011**
EPI records (n = 278)^1^	87	89.6	91.3	88.4	73.6	86	
	(76.7, 93.8)	(78.8, 96.1)	(79.2, 97.6)	(76.6, 95.6)	(59.7, 84.7)	(81.3, 89.8)	0.786
*% of EPI records found*	91%	82%	84%	82%	70%	81%	

^1^ Restricted analysis to children with serum and vaccination card records (n = 294), adequate OF samples (n = 330), or EPI records (n = 278).

^2^ Z-test comparing quintile 1 vs. quintiles 2–5.

### Agreement & performance of MCV1 indicators at individual level

We also assessed within-child agreement for each data source. MCV1 history by OF had poor sensitivity (62.3%; CI: 59.3, 65.3) and specificity (69.4%; CI: 62.4, 75.8) compared to card + history, which was considered the gold standard. Maternal report had high sensitivity (99.4%; CI: 98.7, 99.8) but poor specificity (55.4%; CI: 48.1, 62.6) (Additional file 1: Table S2). To assess agreement between maternal report and card records, the analysis was restricted to children with vaccination cards. 99% of mothers with card-confirmed MCV1 reported MCV1 history. For those without card-confirmed MCV1, 43% still reported MCV1 history, resulting in 91% agreement and a kappa of 0.54 (95% CI: 0.46, 0.62) between vaccination cards and maternal report. 150 children had vaccination cards but lacked evidence of MCV1 receipt on their card. Among the 150, 105 (70%) had a date of MCV1 receipt in the EPI record books, 86 (57%) mothers reported the child received MCV1, and 67 (45%) had both. 43 of the 150 children had blood results, of which 25 (58%) were seropositive and only 18 (42%) were seronegative as expected from their vaccination card.

Similarities in coverage at the population level often masked discordance between indicators. Among children with both vaccination cards and EPI records, MCV1 coverage was the same by EPI record or vaccination card but within-child agreement was only 73.8% (CI: 70.6, 76.8) because an equal number of children were positive by one source and negative by the other (Table 5).

**Table 5:** Discordance between MCV1 history in EPI records & vaccination cards

		***Vaccination card***	
		**MCV1 (+)**	**MCV1 ( − )**
***EPI***	**MCV1 (+)**	694	105
***records***	**MCV1 (−)**	105	1

Note: Includes only children with vaccination card records and EPI clinic records (n = 800).

## Discussion

Using surveys, clinic records and immunologic markers, up to six measures of MCV1 coverage were generated for a representative sample of children in rural Bangladesh. No prior study has compared as many indicators including immune markers from a single cohort of children. Apart from OF results, vaccination coverage estimates did not vary dramatically across data sources or indicators. MCV1 coverage from reported or recorded data ranged from 84% to 91%, with a best estimate of 84% based on the ‘gold standard’ card + history indicator. A 2010 Bangladesh government survey using the card + history indicator found 84% MCV1 coverage for the district, a catchment area slightly larger than the DSS [30].

Immune marker surveillance with OF was not an accurate indicator of vaccination history compared to all other indicators. The proportion of children with detectable antibodies in OF was significantly lower than expected by reported or recorded MCV1 coverage even after adjusting for incomplete seroconversion and assay performance [29]. In the subset of children with blood samples, poor agreement between blood and OF results reinforced that OF testing was not an accurate immune marker and is not recommended for use in immunization surveillance unless the validity and reliability of OF testing can be improved [31].

Our results highlighted that mothers who received antenatal, delivery and postnatal care in the formal health system were more likely to have MCV1 vaccinated children. Unlike some other studies from South Asia, boys were not more likely than girls to be vaccinated with MCV1 [32,33]. Unvaccinated children were more likely to live in households with relatively lower SES and education. After accounting for reported vaccination history, children in the poorest 20% were significantly more likely to be measles susceptible than those in the upper 80%, warranting further investigation into biological or systems-level causes of susceptibility among the very poor [34,35].

Many studies have shown that reported and recorded vaccination history data are fraught with biases and errors in recall and omission [4,6,8,11,16,36,37]. Studies in Bangladesh [38], Costa Rica [9], and England [39] found that maternal report inflated coverage estimates but our results illustrated that maternal report or card + history indicators performed well compared to serological and government estimates of MCV1 coverage. Without a gold standard of vaccination history, it was not possible to assess if maternal report overestimated coverage. What may be more important is to generate vaccination coverage estimates that can be compared across indicators. Currently policymakers frequently compare vaccination coverage estimates generated from different indicators and data sources, resulting in an ‘apples to oranges’ comparison. Table 2 presents an example of how adjustment factors could be generated for each pair of coverage indicators to evaluate the degree of over- or underestimation with each indicator. Because the indicators used in this study were generated in the same way as national coverage estimates from administrative records, coverage evaluation surveys and DHS surveys, a similar table of adjustment factors could be generated from a small representative sample of children in a country and then used to adjust national coverage estimates to make them more comparable between data sources.

Our results called into question whether vaccination card records should be considered the best available information for a child’s vaccination status because missing vaccination records on the card could be verified with other data sources. DHS and MICS surveys seek maternal report of vaccination history only when the child’s vaccination card is not available. Future studies should explore if vaccination history by maternal report should also be used for children who have cards but lack evidence of the vaccination on their card.

Because children with cards were more likely to be vaccinated and their mothers were more likely to correctly recall MCV1 status, survey with higher card retention rates may have less error in coverage estimates. Consequently, we recommend that the card retention rates should always be presented as an indicator of data quality when the card + history indicator is used.

Our results should be interpreted in light of the limitations of the data. Without a vaccination registry, there was no gold standard measures of vaccination history or measles immunity [40]. We used strict criteria for matching children with their EPI clinic records to avoid matching errors, but this may have underestimated the number of children with EPI records and induced a bias that overestimated MCV1 coverage by EPI record. Results from OF testing generated significant concern about its validity for immunization surveillance. Several other studies have shown good sensitivity and specificity for the assay, suggesting that the problems in this study could be ameliorated [41-43].

## Conclusions

This study illustrates that a simple comparison of data can have broad implications for immunization surveillance. Surveys using the card + history or maternal report indicators combine feasibility with accuracy in this setting and continued use is recommended to monitor secular trends for MCV1 in Bangladesh. However, an equally accurate indicator generated in real-time is needed and may be best achieved by replacing EPI record books with electronic records to document a child’s vaccination history wherever they get vaccinated. Given the low refusal rate for blood collection in this study, other countries with inaccurate reported or recorded vaccination indicators should consider serological surveillance for measles antibodies as an alternative or corollary to existing surveillance tools. As countries like Bangladesh shift their focus from measles control to elimination and eradication, serological surveillance to estimate population immunity may become an increasingly important indicator [44-46]. A careful consideration of the costs and logistical challenges of serosurveillance need to be weighed against the potential benefits of using immune markers to monitor immunization systems.

## Abbreviations

CI: Confidence interval; DHS: Demographic and Health Surveys; DSS: Demographic Surveillance System; ELIS: Enzyme linked immunosorbent assay; EPI: Expanded Programme on Immunisation; IgG: Immunoglobulin G; OF: Oral fluid; OR: Odds ratio; MICS: Multiple Indicator Cluster Survey; MCV1: Measles containing vaccine, first dose; SES: Socioeconomic status.

## Competing interests

The authors declare that they have no competing interests.

## Authors’ contributions

KH designed study with input from MS, HAE, DB, WM, and OL. KH and MS managed data collection. HAE managed and WM advised on laboratory procedures. KH conducted analysis and led manuscript preparation with input from MS, HAE, DB, WM, and OL. All authors read and approved the final manuscript.

## Pre-publication history

The pre-publication history for this paper can be accessed here:

http://www.biomedcentral.com/1471-2458/13/1211/prepub

## Supplementary Material

Additional file 1 Supplementary tables and figures. Click here for file
