# Supplementary material for: Measles vaccination coverage estimates from surveys, clinic records, and immune markers in oral fluid and blood: a population-based cross-sectional study
Source: BMC Public Health. 2013 Dec 20;13:1211. doi: 10.1186/1471-2458-13-1211 (PMC3890518; doi:10.1186/1471-2458-13-1211)
Supplement: Additional file 1 — Supplementary tables and figures. [file 1471-2458-13-1211-S1.docx]

**Supplementary Tables and Figures**

Authors: Hayford K, MS Shomik, HM Al-Emran, WJ Moss, D Bishai, and OS Levine.

Title: Measles vaccination coverage estimates from surveys, clinic records, and immune markers in oral fluid and blood: A Population-based cross-sectional study.

**Table S1. Characteristics of Enrolled and Non-Enrolled Children Based on Data from DSS Database**

|  | **Enrolled (n=1259)** | | **Not Enrolled (n=129)** | |  |
| --- | --- | --- | --- | --- | --- |
|  | **Frequency** | **%** | **Frequency** | **%** | ***p-value*** |
| Age at enrollment, in months, mean (SD) | 13.83 (1.38) | | 13.95 (1.42) | | 0.423 |
| Male | 642 | 51.0 | 61 | 47.3 | 0.460 |
| Religion |  |  |  |  |  |
| Muslim | 1115 | 88.6 | 114 | 88.4 | 0.948 |
| Hindu | 144 | 11.4 | 15 | 11.6 |  |
| Household size, median (IQR) | 5 [4, 7] |  | 6 [4, 8] |  | 0.328 |
| Father’s Education^1^ |  |  |  |  |  |
| No formal education | 227 | 18.0 | 18 | 14.0 | 0.802 |
| Primary incomplete | 83 | 6.6 | 7 | 5.4 |  |
| Primary complete | 184 | 14.6 | 20 | 15.5 |  |
| Secondary incomplete | 296 | 23.5 | 35 | 27.1 |  |
| Secondary complete or higher | 193 | 15.3 | 18 | 14.0 |  |
| Missing | 276 | 21.9 | 31 | 24.0 |  |
| Mother’s Education^1^ |  |  |  |  |  |
| No formal education | 175 | 13.9 | 10 | 7.8 | 0.210 |
| Primary incomplete | 102 | 8.1 | 10 | 7.8 |  |
| Primary complete | 232 | 18.4 | 19 | 14.7 |  |
| Secondary incomplete | 592 | 47.0 | 70 | 54.3 |  |
| Secondary complete or higher | 151 | 12.0 | 19 | 14.7 |  |
| Missing | 7 | 0.6 | 1 | 0.8 |  |
| Mother – housewife^1^ | 1187 | 94.3 | 112 | 86.8 | **0.007** |
| Family moved at least once in last 3 years | 498 | 39.6 | 75 | 58.1 | **<0.001** |
| Asset Quintile (2007)^1^ |  |  |  |  |  |
| 1 (poorest) | 256 | 20.3 | 25 | 19.4 | 0.249 |
| 2 | 254 | 20.2 | 28 | 21.7 |  |
| 3 | 261 | 20.7 | 17 | 13.2 |  |
| 4 | 248 | 19.7 | 27 | 20.9 |  |
| 5 (wealthiest) | 234 | 18.6 | 31 | 24.0 |  |
| Missing | 6 | 0.5 | 1 | 0.8 |  |
| ^1^ Data generated from DSS database. Education, employment and asset quintile were collected in 2007 or upon migration into the DSS area. | | | | | |

**Table S2. Comparison of Sensitivity, Specificity, PPV, NPV and Kappa for Vaccination Coverage Indicators in Study.**

| **Performance Compared to Card + Report (DHS)** | | | | | |  |  |  |  |
| --- | --- | --- | --- | --- | --- | --- | --- | --- | --- |
| *Gold Std:  Card + History* | **N** | | **Sensitivity** | | **Specificity** | **PPV** | **NPV** | **Concor-dance** | **Kappa** |
| Maternal report^1^ | 1226 | | 99.4  [98.7, 99.8] | | 55.4 [48.1, 62.6] | 92.3  [90.5, 93.8] | 94.7  [88.8, 98.0] | 92.5% | 0.66 [0.60, 0.72] |
| Oral fluid | 1226 | | 62.3 [59.3, 65.3] | | 69.4 [62.4, 75.8] | 91.6  [89.3, 93.5] | 25.6  [21.9, 29.6] | 63.5% | 0.19 [0.14, 0.24] |
| EPI record | 891 | | 86.9  [84.3, 89.2] | | n/a^2^ | 84.8  [82.1, 87.2] | n/a^2^ | 75.2% | -0.13 [-0.16, -0.10] |
| Serum | 330 | | 95.0 [91.6, 97.3] | | 48.0 [33.7, 62.6] | 90.5 [86.4, 93.7] | 64.9 [47.5, 79.8] | 87.5% | 0.48 [0.34, 0.62] |
| ^1^ For 17% of children without vaccination cards, DHS vaccination status is based on maternal report and therefore 100% concordant. | | | | | | | | | |
| ^2^ Due to small sample size in the numerator, specificity and NPV not presented and kappa and c-statistic should be interpreted with caution. 76 unvaccinated children by card+history could not be found in EPI records | | | | | | | | | |
|  | |  | |  |  |  |  |  |  |
| **Performance compared to Serum** | | | | |  |  |  |  |  |
|  | **N^1^** | | **Sensitivity** | | **Specificity** | **PPV** | **NPV** | **Concor-dance** | **Kappa** |
| Maternal report | 311 | | 98.5  [96.3, 99.6] | | 64.9 [47.5, 79.8] | 95.4 [92.3, 97.5] | 85.7 [67.3, 96.0] | 94.5% | 0.71 [0.58, 0.84] |
| Card + History | 311 | | 90.5  [86.4, 93.7] | | 64.9 [47.5, 79.8] | 95  [91.6, 97.3] | 48.0 [33.7, 62.6] | 87.5% | 0.48  [0.34, 0.62] |
| Oral fluid | 311 | | 60.2 [54.2, 66.1] | | 75.7 [58.8, 88.2] | 94.8 [90.4, 97.6] | 20.4 [14.0, 28.2] | 62.1% | 0.16  [0.08, 0.25] |
| EPI Records | 251 | | 85.4 [80.1, 89.7] | | 4.0  [ 0.1, 20.4] | 88.9  [84.0, 92.8] | 2.9 [ 0.1, 15.3] | 77.3% | -0.09 [-0.17, -0.01] |
| ^1^ Restricted analysis to children with adequate oral fluid specimen and serum specimen. 251 of 311 children also had matched EPI records. | | | | | | | | | |
